# NS-GUSL: Green U-Shaped Learning for Nuclei Segmentation from Histopathology Images

**DOI:** 10.3390/jimaging12070316

**Published:** 2026-07-10

**Authors:** Catherine Aurelia Christie Alexander, Vasileios Magoulianitis, Jiaxin Yang, C.-C. Jay Kuo

**Affiliations:** 1 Ming Hsieh Department of Electrical and Computer Engineering, University of Southern California (USC), Los Angeles, CA 90089, USA; 2 Department of Urology, Keck School of Medicine, University of Southern California (USC), Los Angeles, CA 90033, USA

**Keywords:** nuclei segmentation, histopathology images, machine learning, low-confidence samples, green learning

## Abstract

Nuclei segmentation is a key task in digital histopathology, highlighting important aspects of nuclear morphology and topology in many cancer-related evaluations and studies. Variability in nuclear appearance both within and across different organs, stain heterogeneity, and inconsistencies in acquisition procedures contribute to the complexity of the task. The existing nuclei segmentation methods apply deep learning to address these challenges, using models with millions of parameters, thereby significantly increasing computational complexity. They also face limitations in generalizing to unseen organs and slide preparations. In this paper, we propose a transparent and lightweight Green U-Shaped Learning model for nuclei segmentation (NS-GUSL). NS-GUSL features a multi-scale architecture for coarse-to-fine refinement of probability maps, which are subsequently binarized using a novel low-confidence sample binarization (LCSB) technique. The model features a modular, feed-forward feature learning scheme with unsupervised representation learning and supervised feature selection and generation. A final morphological post-processing step refines the segmentation maps to improve instance separation while preserving nuclei convexity. The model was trained and tested on the MoNuSeg dataset and compared against other deep learning baselines for segmentation performance. In addition, external validation experiments were conducted to evaluate the proposed model’s generalizability to unseen organs and staining procedures. NS-GUSL exhibits the best panoptic segmentation performance and competitive detection quality across all datasets. Moreover, our model is shown to be compact, low in computational complexity, and to have a minimal carbon footprint, compared to other deep learning models, making it a suitable choice for deployment on edge devices.

## 1. Introduction

Histopathological evaluation is a critical step in the assessment of many diseases, including cancer. It involves studying nuclear morphology and topology, which are key indicators of tumor grade, cancer subtypes, and disease progression [1]. Traditionally, pathologists analyzed biopsied tissue slides under high-power microscopes to make informed clinical decisions, including diagnosis, tumor grading, prognosis, and the development of a customized treatment plan [2,3].

Tissue slides are prepared for evaluation by staining with hematoxylin and eosin (H&E) to highlight the nuclei regions. The stained slides are subject to variations that stem from non-standard staining protocols, introducing artifacts that increase nuclei variability across slides and scan centers [4]. The advent of digital scanners has enabled the digitization of histopathology slides, now known as whole-slide images (WSIs). Differences in digitization methods between scanners introduce variations in nuclei color and texture [5]. In addition, nuclear morphology, density, and distribution vary between organ tissues, further complicating the task.

The analysis of high-resolution WSIs for nuclear morphology and texture is a labor-intensive and time-consuming task. In addition, differences in levels of expertise can lead to subjective evaluations of these images. The development of AI tools for nuclei segmentation considerably simplifies and accelerates histopathological analysis by providing objective decisions and highlighting the essential features of nuclei for cancer profiling [6].

Traditional nuclei segmentation methods, such as thresholding, level sets, and graph cuts, face several challenges. They require manual tuning of hyperparameters, have limited generalizability [7], and struggle to resolve difficult or confusing cases [8]. The development of deep learning (DL) models has shown promising results in this task, with their ability to map inputs to outputs by learning complex patterns from the image. Convolutional neural networks, such as Fully Convolutional Networks (FCNs) [9] and Regional Convolutional Neural Networks (RCNNs) [10,11], were adapted to the medical image domain. In addition, U-Net [12] was developed with a multi-scale encoder-decoder architecture, specifically for biomedical image segmentation. Many advanced U-Net-based methods [13,14,15,16] have been proposed for nuclei segmentation, achieving remarkable results. Many researchers continue to build on these foundations by addressing challenges such as stain heterogeneity and boundary delineation.

Although DL models exhibit good performance, there are some challenges when applied to the healthcare domain. Most existing DL models perform supervised nuclei segmentation using pixel-level annotations and require large amounts of labeled data. Due to the large size and high resolution of these WSIs, obtaining annotations is expensive and difficult [17,18]. Given the limited availability of medical image data, training complex DL models with limited labeled data can affect their generalizability to images from other laboratories, patient demographics, and scanners [18,19]. Furthermore, low annotation precision can degrade the AI model’s performance. Deep learning models are often viewed as “black boxes” by clinicians due to their lack of transparency in decision-making [20]. The limited interpretability restricts the adoption of such models in the real world. In addition, DL models are computationally intensive and require expensive resources for clinical deployment.

To address these challenges, this paper introduces a Green U-Shaped Learning Model for Nuclei Segmentation (NS-GUSL) based on the Green Learning framework. Green Learning uses a feed-forward mechanism for representation learning with a transparent, modular architecture. NS-GUSL is a four-level regression model for 2D medical image segmentation. The main contributions of this paper are summarized as follows:

- We propose a 2D Green U-Shaped Learning framework for nuclei segmentation (NS-GUSL), featuring a small model size and low computational complexity.
- A low-confidence sample binarization (LCSB) technique is implemented to improve the predictions of hard samples, such as outlier nuclei or boundary regions.
- This work demonstrates competitive quantitative and qualitative performance in various metrics.
- We present a cross-dataset validation study to demonstrate the generalizability of our model across datasets compiled under different conditions.

## 2. Related Work

### 2.1. Traditional and Machine Learning Methods

Early works applied various combinations of image processing techniques to segment nuclei in histopathological images. Thresholding aims to isolate nuclei from the background, generally based on image histograms [21,22,23]. Active contours [24] and level sets [25] focus on the evolution of the boundary through curves and functions, respectively. To split connected nuclei instances, the watershed algorithm gradually expands regions from a starting seed, usually an approximation of nuclei centers [26,27]. In addition, traditional approaches use priors on nuclear size, shape, and texture to make decisions in multiple stages without any supervision. Despite achieving reasonable performance, these methods lack robustness to stain and lighting heterogeneities and require manual hyperparameter tuning, affecting generalizability.

Supervised methods emerged with the development of machine learning (ML). Popular hand-crafted features for ML are those that capture nuclear texture, shape, and intensity from images [28,29]. Selecting appropriate features requires a high level of domain expertise. ML models trained on these features are highly tailored to a specific dataset, sensitive to staining variations, and suffer from limited generalizability [30]. Additionally, accurate boundary delineation of clustered or overlapping nuclei posed a significant challenge.

### 2.2. Deep Learning Methods

The encouraging performance of deep learning in computer vision tasks led to its adoption in medical imaging. It automatically captures meaningful features for a task using end-to-end optimized learning. HoVerNet [31] adopts a customized convolutional neural network (CNN) solution for nuclei segmentation and classification. It comprises a ResNet-50-based encoder and three decoder branches: the first to predict nuclear pixel locations, the second to predict horizontal and vertical distances, and the third to predict nuclear type. Distance maps help to separate clustered and overlapping nuclei, thereby enhancing the predictions of nuclear boundaries.

U-Net [12], originally developed for biomedical image segmentation, has shown promising results for various segmentation tasks. It has been applied to nuclei segmentation with numerous variations, including changes in depth, encoder backbones, attention mechanisms, pooling mechanisms, and more. A key advantage lies in its multi-scale architecture with skip connections between the encoder and decoder at each level that preserve spatial information lost during pooling. U-Net++ [32] moves forward with an ensemble of U-Nets of varying depths and redesigned skip connections via dense multi-scale fusion. It has been shown to outperform fixed-depth U-Net models in several tasks. To compensate for the limited long-range and global semantic learning of convolutions, SwinU-Net [33] uses a pure Transformer-based U-shaped network, where both the encoder and decoder consist of double Swin Transformer blocks. SwinU-Net is shown to surpass the segmentation performance of pure convolutional and convolution-transformer methods. Although pretrained backbones and attention mechanisms further improve segmentation performance, they incur increased complexity and resource consumption.

Accurate delineation of nuclear boundaries remains challenging due to variations in staining and the presence of densely packed nuclei. To focus on boundary regions, Oda et al. proposed a U-Net-based Boundary-Enhanced Segmentation Network (BESNet) [34], with two decoding paths. The boundary decoding path (BDP) is trained on boundary annotations to estimate the confidence of boundary pixels, and the main decoding path (MDP) leverages the feature maps generated by the BDP to guide cell segmentation. To further enhance the performance, the Contour-Aware Information Aggregation Network (CIA-Net) [35] exploits the mutual dependencies between nuclei and contours through the multilevel Information Aggregation Module (IAM). Similarly, the Dual Decoder U-Net [13] uses decoding branches to predict the nuclei and their distance maps, which are then integrated through the watershed algorithm to obtain the final segmentation maps.

TransNuSeg [36] explores the capabilities of pure transformer-based architectures for nuclei segmentation. This work proposes a single encoder and three decoders for nuclei prediction, edge prediction, and clustered edge prediction, respectively, demonstrating the effectiveness of their model in predicting the boundaries of nuclei in densely clustered regions among several other methods. Du et al. [37] propose the Multi-Scale Transformer Attention Module (MSTA), which improves the transfer of contour features from the encoder to decoder, thereby overcoming challenges from blurred cell boundaries and improving boundary segmentation. While these methods improve performance, they require considerably larger models.

Staining heterogeneity among laboratories and patient populations is another challenge that undermines the generalizability of nuclei segmentation algorithms. Ivanov et al. show in ref. [38] that aggregating multiple reference images during stain normalization can enhance the robustness of DL models to staining variations. In ref. [39], Mahbod et al. incorporate test-time stain normalization, where each image is normalized with multiple reference images, and their results are aggregated through weighted averages to enhance the generalization performance. However, the increased performance is achieved at the expense of additional computational overhead.

### 2.3. Green Learning Methods

Introduced by Kuo et al. in ref. [40], the Green Learning framework uses a modular, interpretable, low-complexity approach, with compact, energy-efficient models. Compared to features learned through backpropagation in DL, GL utilizes feed-forward representation learning. This is done using the Subspace approximation with adjusted bias (Saab) transform [41], which extracts rich spatial–spectral representations, yielding lightweight models with minimal complexity.

Green Learning has been applied to several medical imaging tasks and has demonstrated competitive performance compared to DL methods. Ref. [42] was the first method to adopt GL and create PCA-RadHop for clinically significant prostate cancer segmentation. Drawing inspiration from U-Net, a multi-resolution 3D Green U-shaped Learning (GUSL) model [43] was applied to the prostate gland and zonal segmentation. This model generates an initial prediction map and iteratively refines the segmentation maps from coarse to fine resolutions with multiple levels of residue correction. The 3D GUSL model achieved state-of-the-art results among other DL methods. LG-NuSegHop [44] is a self-supervised nuclei segmentation pipeline with the GL-based NuSegHop as a bridge between local and global processing modules. It achieves good generalization performance with minimum parameters.

Motivated by GL’s strong performance in medical image segmentation, our model adopts GUSL for nuclei segmentation and addresses existing challenges through preprocessing, hard sample mining, and post-processing. The proposed NS-GUSL architecture differs significantly from LG-NuSegHop; nevertheless, both models use the Saab transform in forward feature learning.

## 3. Methodology

### 3.1. Preprocessing

**Figure 1:**
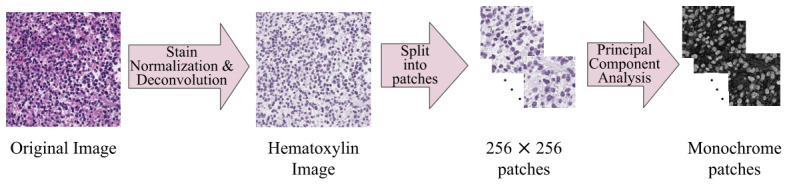
Preprocessing: Each image is stain-normalized and deconvolved to split into hematoxylin (H) and eosin (E) components. The H-image is split into 256×256 sized patches and converted to monochrome through principal component analysis (PCA).

As depicted in Figure 1, the hematoxylin (H) component of the H&E stain colors the nuclei in purplish blue, while the eosin (E) component stains the remaining cellular components in pink. Macenko stain normalization [45] is applied by projecting each image onto a reference image color space to overcome staining variations. The stain-normalized image is deconvolved to separate the H component from the E component, thereby unmixing any overlapping components and improving the contrast between nuclei and background. This H-image is divided into patches of size 256×256 to effectively manage memory. These RGB H-image patches are converted to monochrome by applying principal component analysis (PCA) across the three color channels, and selecting the first principal component, which achieves 99% energy compaction. This operation essentially reduces the number of color channels while preserving important structural and color information. The monochrome patches are processed through NS-GUSL to obtain binary segmentation maps.

### 3.2. NS-GUSL

**Figure 2:**
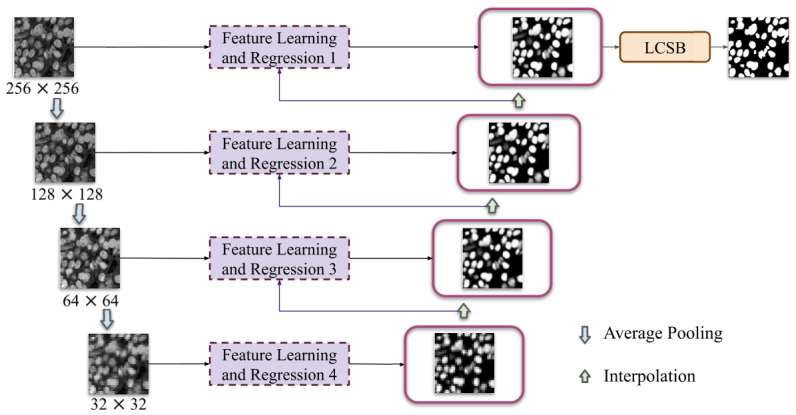
A high-level overview of the multi-scale architecture of the NS-GUSL model for nuclei segmentation. After an initial prediction at the coarsest scale, the predictions are successively refined at each level. The soft predictions at the finest level are binarized by the LCSB module.

The proposed NS-GUSL model, shown in Figure 2, is a four-scale segmentation framework that performs segmentation from coarse to fine resolution. At each level, we generate a probability heatmap that indicates the likelihood that each pixel belongs to the nuclei class; while the images at level 1 are at the highest (original) resolution, the images are average-pooled by a factor of $2^{i-1}$ at levels i>1.

**Figure 3:**
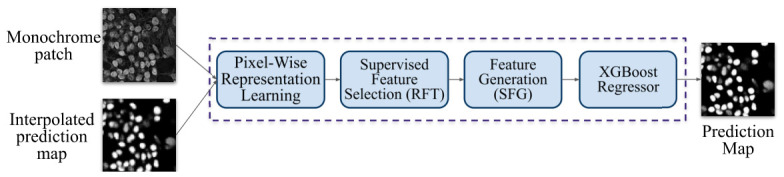
Feature Learning and Regression Module: The inputs are processed through the representation learning module, followed by RFT and SFG for discriminant feature selection and feature generation, respectively. The raw and generated features are concatenated and fed to an XGBoost Regressor to predict the probability of each pixel being a nucleus.

At level 4, we predict an initial probability map $P_{4}$ that we iteratively refine at subsequent levels. The input to the feature learning and regression module 4 is the preprocessed 32×32 monochrome image, denoted by $X_{4}$. Spectral features are extracted from this image using the Saab transform, which computes the spectral components of a pixel neighborhood via principal component analysis (PCA). These spectral features are concatenated with spatial features (i.e., pixel values) and Laws features to capture the texture of nuclei and their boundary regions. At the finer levels 3, 2, and 1, the feature learning and regression module (see Figure 3) has a dual input: one from the downsized monochrome image $X_{i}$ and the other from the interpolated prediction map $P_{i}^{p}$ from the previous level i+1. By doing so, we take into account both the current scale’s feature map and the coarse prediction map from the previous level (scale). Similar to level 4, all the spectral, spatial, and Laws features are learned from the monochrome image. Since the interpolated prediction map is a low-resolution estimate of the segmentation map, it is not expected to contain any textures, and we only learn the spectral and spatial representations for $P_{i}^{p}$.

Because representation learning is unsupervised, many extracted features may not have significant information for this task. Therefore, the learned pixel-wise representations are filtered using feature selection to remove noisy features. To enrich the feature space, the selected set of features (referred to as raw features) is used to generate more powerful features using statistics-based feature generation (SFG). The newly generated features, along with the selected raw features, are used to train the regressor. Detailed explanations about the feature learning process are presented in Section 3.3 and Section 3.4. The XGBoost regressor is trained on average-pooled or original ground-truth labels, depending on the level. The output at level 1 of NS-GUSL is a probability heatmap with original resolution.

### 3.3. Representation Learning

**Figure 4:**
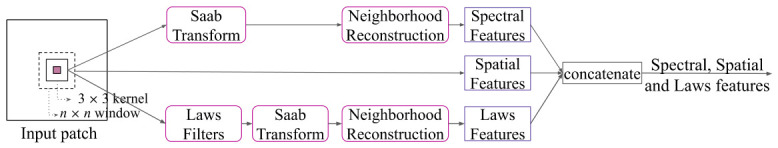
Representation Learning: Spectral, spatial, and Laws features are obtained for each pixel from an n×n neighborhood. The concatenated features constitute the raw features for each pixel. All three branches are used for a monochrome image input, while only the spectral and spatial branches are used for the interpolated prediction input.

Representations are learned in a feed-forward, unsupervised manner in the Green Learning paradigm. For each pixel, we extract three feature sets based on its local neighborhood. This includes features from the Saab transform, spatial features, and texture features computed from the Laws filters. An overview of the spectral and spatial feature extraction process is shown in Figure 4.

#### 3.3.1. Saab Features

The spectral representations for each pixel in the monochrome image were obtained with the help of the Saab transform [41]. It is a statistics-based transformation that takes advantage of the principal component analysis (PCA), and can be expressed as:

(1) $$y_{m}=a_{m}^{T}\cdot x+b_{m},m=0,1,\ldots ,M-1$$

where x$\in R^{k\times k}$ is the input vector, $a_{m}$ is the *m*-th anchor vector, $b_{m}$ is the bias term derived as explained in [41], and *M* is the total number of anchor vectors. The input subspaces can be expressed as a direct sum of the AC and DC subspaces as

(2) $$S=S_{DC}⊕S_{AC}$$

where $S_{DC}$ is spanned by the DC anchor vector, $a_{0}=\frac{1}{\sqrt{N}}{\left(1,1,1,\ldots ,1\right)}^{T}$, and $S_{AC}$ is spanned by the AC anchor vectors, $a_{m}$. Input x is projected onto the DC subspace to get the DC component, $x_{DC}$. To get the AC component, the DC component is subtracted from the input, i.e., $x_{AC}=x-x_{DC}$. PCA is then applied to this AC component to learn the AC anchor vectors $a_{m}$. The input data is then projected onto these learned anchor vectors to obtain spectral representations as defined by Equation (1).

In our model, we use a standard kernel size of 3×3 across all levels. This gives 9 AC kernels and a DC kernel from the local patch mean. A small kernel size pays attention to more local detail while minimizing computational complexity. To capture richer contextual information, we include the transform coefficients of pixels in the neighborhood. The size of the neighborhood is set as shown in Table 1. Typically, we increase the size of the neighborhood at finer levels to capture sufficient spatial information.

#### 3.3.2. Spatial Features

To learn spatial representations in addition to spectral components, we collected pixel values from a local neighborhood around each pixel. The size of the local neighborhood is set to match the overall window size used for spectral feature extraction. Finer levels use larger neighborhoods to encompass more contextual information in these resolutions. In addition, we learn representations of the local binary pattern in the pixel neighborhood to include intensity-independent texture information.

#### 3.3.3. Laws Features

The nuclei contain a material called chromatin, whose texture differs from that of other cellular components. This information can be a key factor that distinguishes between nuclei and background regions. Laws filters are a popular image processing technique for observing textural information. These filters are very helpful for detecting localized texture patterns, such as edges, and for distinguishing between nuclei and background regions. In our work, we use three of the 3×3 Laws filters, namely $L_{3}E_{3}$, $E_{3}L_{3}$, and $E_{3}E_{3}$, to capture horizontal edges, vertical edges, and high-frequency textures. After obtaining the texture map for each filter, we apply the Saab transform with a 3×3 kernel to each map to learn the texture representation for each pixel. Similar to learning spectral representation, we apply neighborhood reconstruction to obtain representations from local neighborhoods. The window size chosen at each level is shown in Table 1. Including these features, along with the spectral representations of the monochrome image, helps focus on edge information and its orientations, thereby improving boundary learning.

### 3.4. Feature Selection and Learning

Up until this step, we do not involve labels in representation learning. The next module performs feature selection using labels to eliminate noisy features that risk overfitting and increase computational complexity. The Relevant Feature Test (RFT) and Discriminant Feature Test (DFT) [46] are used to select useful features in regression and classification tasks, respectively. These supervised feature selection methods use training labels to assess the discriminative power of each feature dimension. The subspace for each feature dimension is partitioned using an ideal threshold that minimizes loss. This loss is selected to be the total mean squared error for RFT and the weighted entropy loss for DFT. The loss value for each feature dimension indicates its discriminant power. The lower loss suggests a higher discriminant power of a feature for a given set of samples. Joint training–validation assessment is applied to select *k* features with minimum loss values from both sets.

To further enrich the feature space for the nuclei segmentation task, we employ a supervised statistics-based feature generation (SFG) method [47]. Although selected raw features are analyzed for their discriminative power, it is possible to generate more powerful features through the Least-Squares Normal Transform (LNT) method [48]. Firstly, a depth-1 auxiliary XGBoost model is used to select a subset of raw features tailored to a target subspace. An LNT kernel is defined as the set of features selected to generate a new feature. In this model, we use variable LNT kernel sizes ranging from 2 to half the size of the raw feature subset. A linear regressor is trained on each LNT kernel to compute weights that are used to linearly combine the features in the LNT kernel and generate one new feature. The LNT features generated from the different LNT kernels are generally more powerful than the raw features, with lower loss values. The generated LNT features are generally more powerful than the raw features, with lower loss values. We perform a second round of feature selection to eliminate noisy LNT features. Through experiments on a validation set, it was observed that concatenating the raw and LNT features showed improved performance than using either the raw or the LNT features alone. Based on this observation, the concatenated raw and LNT features are used in the regression tasks. This process is executed once at each level of the NS-GUSL model.

**Figure 5:**
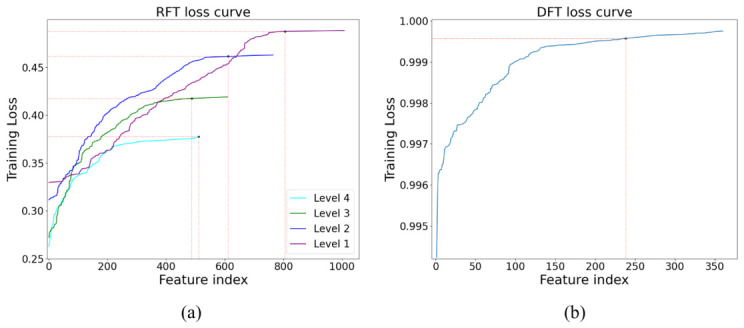
Loss curves used for feature selection at each level. Features within the red dotted lines are selected as final XGBoost features. (**a**) RFT loss curves for Levels 4, 3, 2, and 1; (**b**) DFT loss curve for LCSB.

Table 2 outlines the number of raw features, RFT/DFT selected features, and the LNT generated features. We apply feature selection once again after concatenating the selected raw and LNT features to get the final features for XGBoost. After ranking the features at each level, we empirically select the number of features with the help of a validation set. The loss curves for this final feature selection at each level is depicted in Figure 5. All features that fall within the dotted red lines are selected as the XGBoost features for that level.

### 3.5. Regression

At each level, NS-GUSL uses the XGBoost regressor to predict the probability that each pixel belongs to the nucleus class. The regression targets at levels 4, 3, and 2 are the average-pooled values of ground truth in [0, 1]. Level 1, the original resolution, has ground-truth values 0 and 1. However, we still apply the regressor to predict the probability of pixel here. All four regressors are trained with the logistic regression objective to restrict predicted values to the range [0, 1], mimicking probabilities.

### 3.6. Hard Sample-Aware Training

While a majority of the samples can be predicted with a small or negligible error, the dataset also contains a limited number of difficult samples that are often predicted with a very high error rate (e.g., nuclei boundaries). However, regular XGBoost training treats all samples equally and does not account for their difficulty. During NS-GUSL training, we apply two techniques to improve the representation of hard samples in the training distribution.

The first technique involves analyzing the original data distribution to identify samples with large errors and constructing a balanced training dataset comprising easy and sufficient hard samples. To compensate for the lack of prior knowledge of sample difficulty at level 4, we train an auxiliary XGBoost regressor on the original data without any augmentation. We compute the magnitude of the residues (the difference between ground truth and predictions) to identify hard samples based on the confidence distribution of the regressor. The threshold for distinguishing between easy and hard samples is empirically selected to be the median value of the absolute residues from the training distribution. During actual XGBoost regressor training, we augment hard samples using the techniques described in Section 4.2 to increase their frequency in the training distribution, thereby balancing the easy and hard samples. Note that the auxiliary XGBoost is used only to identify hard samples and is not part of the inference. At levels 3, 2, and 1, instead of an auxiliary XGBoost regressor, we leverage the interpolated probability maps from the previous level to identify and balance the easy and hard samples during training, using a similar easy–hard threshold selection strategy as level 4. This process allows the model to see more hard examples during training and helps reduce their errors. In addition, we apply gradient-based sampling in the XGBoost regressor, so that the samples with larger errors have a higher probability of being selected at each boosting iteration.

### 3.7. Low-Confidence Sample Binarization (LCSB)

After level 1, we obtain a heatmap of the nuclei probabilities. The next step is to convert this heatmap to a binary segmentation map, while thresholding is a simple solution, selecting the threshold is critical. Although 0.5 is the most common choice, this thresholding neglects the class imbalance and may therefore not be optimal for medical images. In addition, strict thresholding reduces the generalizability of the model. To improve the robustness of the model, we apply a learning-based binarization, particularly for samples with probabilities in the range (0.4, 0.6). Most of the samples in this range are from boundary regions, while some are from hard instances. Such samples are often referred to as low-confidence predictions because a slight adjustment of the threshold can change the final sample prediction. Therefore, we propose a low-confidence sample binarization technique to improve the prediction confidence of these samples. To evaluate the sensitivity of the proposed LCSB module to the low-confidence sample probability thresholds, additional experiments were conducted using several threshold ranges. The corresponding settings are summarized in Section 5.3.

**Figure 6:**
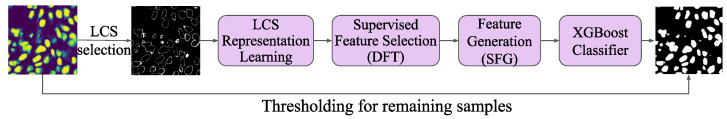
Low-Confidence Sample Binarization (LCSB): For low-confidence samples (LCS), representation learning is followed by DFT for feature selection and SFG for feature generation. An XGBoost classifier trained on the learned features predicts the binary label for LCS, and thresholding is applied to binarize the remaining samples’ predictions.

The low-confidence sample binarization (LCSB) method is depicted in Figure 6. First, samples with prediction probabilities in the range (0.4, 0.6) are identified. All representations for these samples are taken directly from the monochrome image. Similar to representation learning in the NS-GUSL model, we obtain spectral features with a Saab kernel size of 11×11, spatial features, and Laws features with a 3×3 Saab kernel from a 11×11 neighborhood. To expand the feature space, we add local binary pattern features at two scales, Haralick texture features (from [49]), and Histogram of Oriented Gradient (HOG) features. These additional features help in tracking subtle texture transitions, regional statistics, and boundary orientations, respectively. The Discriminant Feature Test (DFT) is then used to select the most discriminant features from the collected raw features, followed by LNT to generate more powerful features. The combined raw and LNT features are used to train an XGBoost binary classifier to predict the label of the selected samples. For the remaining samples with prediction probabilities *p* less than 0.4 and greater than 0.6, the labels are assigned as follows:

(3) $$\begin{array}{c} 0,\;\;\mathrm{if}\;p<0.4\\ 1,\;\;\mathrm{if}\;p>0.6 \end{array}$$

Since this is a pixel-wise operation and is prone to noisy, isolated predictions, we follow this module with morphological post-processing to refine the segmentation map.

### 3.8. Post-Processing

To reduce segmentation noise and enhance the segmentation quality, we apply morphological post-processing. Small spurious predictions resulting from the LCSB module are filtered out to smoothen the segmentation map. Due to textural inconsistencies, there may be holes within a convex-shaped prediction, hinting at the presence of a nucleus. Hole filling is performed to close these convex predictions. With prior knowledge of nuclei’s size and shape, we applied the convex hull algorithm to split overlapping and clustered nuclei. Although these instances have already been detected, this step improves instance segmentation and alleviates under-segmentation and over-segmentation.

## 4. Experimental Setup

### 4.1. Datasets

#### 4.1.1. MoNuSeg

The Multi-Organ Nuclei Segmentation (MoNuSeg) dataset [7] consists of 30 hematoxylin and eosin-stained training images of size 1000×1000, with 21,623 annotated nuclei. It includes H&E images at a magnification of 40×, from seven organs: breast, kidney, liver, prostate, bladder, colon, and stomach. MoNuSeg also comprises a test set of 14 images, including lung and brain images, as well as images of the other seven organs. The H&E images come from 18 different hospitals, which introduce variations into the staining and acquisition methods. The diversity and richness of this dataset make it a good choice to evaluate the generalizability of any segmentation model. Each image is split into 16 overlapping patches of size 256×256 for processing by the NS-GUSL model. Our NS-GUSL model is trained using the MoNuSeg training set and evaluated using the MoNuSeg test set. During inference, the patches are stitched together by averaging the probability values in the overlapping areas.

#### 4.1.2. CPM-17

The CPM-17 dataset [50] contains 32 training and 32 testing H&E images of four cancer types, collected as part of the Computational Precision Medicine Digital Pathology Challenge in 2017. It contains 7570 annotated nuclei with images varying in size from 500×500 to 600×600 and magnifications 20× and 40×. For simplicity, we resize them all to 512×512 and split them into 256×256-sized patches for processing. The different magnifications help us evaluate the generalizability of our model to different scales.

#### 4.1.3. CryoNuSeg

The Cryosectioned Nuclei Segmentation (CryoNuSeg) dataset [51] is the first dataset derived from frozen H&E tissue samples. The overall appearance of the nuclei in this dataset differs slightly due to variations in tissue preparation, making it more challenging and useful to evaluate the robustness of our model. This dataset contains 30 images of size 512×512. The images include H&E slides at 40× magnification from 10 organs, including the larynx gland, adrenal, lymph nodes, pancreas, skin, pleura, mediastinum, thyroid gland, thymus, and testes. Each image is split into four 256×256-sized patches for processing using the NS-GUSL model.

#### 4.1.4. TNBC

The Triple-Negative Breast Cancer (TNBC) Dataset [52] was curated at the Curie Institute and contains 50 H&E images from 11 WSIs with TNBC. These images show a good inter- and intra-patient diversity for this type of cancer. Each image is 512×512 at 40× magnification and contains 4056 annotated nuclei across the entire dataset. The slides are produced using a different preservation and staining technique, making them a suitable choice for a cross-dataset generalization study. The images are split into four 256×256-sized patches for processing through our model.

### 4.2. Data Augmentation

We apply two types of data augmentation to increase the diversity of our training samples. Geometrical transformations, including random rotations and flipping, are performed to generate image patches with different orientations. These transformations help the model be invariant to changes in orientation or spatial position. In addition, we apply color-based augmentations, including brightness shifts, contrast adjustments, gamma correction, and Gaussian blurring. This helps the model to be robust to different types of staining variation observed in H&E images, thus improving generalizability.

### 4.3. Evaluation Metrics

The Aggregated Jaccard Index (AJI) is an advanced version of the Jaccard Index, introduced as a metric to evaluate the performance of nuclei segmentation [7]. The AJI computes the aggregated intersection cardinality in the numerator and the aggregated union cardinality in the denominator for the ground truth and predicted nuclei, including unmatched predicted instances. In this way, false positives and false negatives are penalized in the denominator. Hence, the AJI ensures that all kinds of error are accounted for, including missed detections, false detections, under-segmentation, and over-segmentation errors. In Equation (4), *N* refers to the set of false positives from the prediction set, $G_{i}$ refers to the ground-truth instance, and $P_{i}$ refers to its corresponding predicted instance.

(4) $$AJI=\frac{\Sigma_{i=1}^{n}G_{i}\cap P_{i}}{\Sigma_{i=1}^{n}G_{i}\cup P_{i}+\Sigma_{k\in N}P_{k}}.$$

To demonstrate the robustness of our model, we compare our method with other detection and segmentation metrics. The F1 score, widely used in image segmentation tasks, is a good indicator of instance-level detection, but does not strictly penalize segmentation errors. It is computed as follows:

(5) $$F1\text{-}score=\frac{2TP}{2TP+FP+FN}$$

The Dice coefficient is another popular metric that evaluates segmentation performance by measuring pixel-level overlap between the ground-truth and predicted masks. It is calculated as shown in Equation (6).

(6) $$Dice=\frac{2(X\cap Y)}{\left|X|+|Y\right|}$$

Panoptic quality (PQ) measures the performance of instance-level segmentation through a joint assessment of detection and segmentation. PQ is defined as the product of detection quality (DQ) and segmentation quality (SQ), where DQ is the scaled F1 score, and SQ measures how well true positives match their ground-truth masks.

(7) $$PQ=\frac{\left|TP\right|}{\left|TP\right|+\frac{1}{2}\left|FP\right|+\frac{1}{2}\left|FN\right|}\times \frac{\Sigma_{(x,y)\in TP}IoU\left(x,y\right)}{\left|TP\right|}=DQ\times SQ$$

Here, TP denotes the true positives, FP denotes the false positives, FN denotes the false negatives, and IoU refers to the Intersection over Union score that evaluates the shape concordance between the predictions and ground truth.

## 5. Experimental Results

### 5.1. Quantitative Results

Table 3 compares the performance of our NS-GUSL model with other deep learning frameworks, including U-Net, U-Net++ [53], SwinU-Net, CMF-UNet [54], UCTransNet [55], and Offline-Normalized DDU-Net [39], on the MoNuSeg test set. The pretrained U-Net, U-Net++, and SwinU-Net models were finetuned for the MoNuSeg dataset before evaluation.

Compared to baseline U-Net, U-Net++, SwinU-Net, CMF-UNet, and UCTransNet, NS-GUSL has superior detection performance, as indicated by its high F1 and PQ scores. It also achieves competitive performance on the Dice objective and the AJI, suggesting strong segmentation performance and instance separation on the MoNuSeg test set. Although CMF-UNet achieves a slightly higher AJI than NS-GUSL, its substantially lower F1 score suggests that while it performs well in instance segmentation, its nuclei detection capability is comparatively weaker. While ON-DDU-Net performs better in terms of AJI and Dice, its significantly lower PQ compared with NS-GUSL indicates that it excels at segmentation quality but is less effective at instance detection. These competitive results highlight the ability of NS-GUSL to perform well with purely domain-specific training on a small dataset in contrast to deep learning models pretrained with ImageNet and further fine-tuned on domain-specific data. This is attributed to the statistics-based feature extractor, the Saab transform, that can efficiently learn representations from a small amount of data.

To evaluate the robustness and generalizability of NS-GUSL, we studied the cross-dataset generalization of our MoNuSeg-trained NS-GUSL applied to three other datasets: CryoNuSeg (Table 4), CPM-17 (Table 5), and TNBC (Table 6). Across most datasets and metrics, our NS-GUSL model achieves performance comparable to or better than U-Net, a standard baseline for image segmentation models.

The images in the CryoNuSeg dataset use a different slide preparation method than those in MoNuSeg. In this dataset, NS-GUSL outperforms all DL methods in the PQ index, which jointly assesses detection and segmentation quality. Although other models achieve better performance in the AJI and Dice metrics, NS-GUSL achieves comparable performance in the F1 score and PQ metric. This suggests that our model is efficient at detecting instances, while boundary segmentation remains an area for improvement. The CPM-17 dataset consists of images that are very similar in appearance to those in MoNuSeg, but come purely from cancerous tissue. In this dataset, NS-GUSL outperforms DL baselines in F1 and PQ metrics, indicating strong detection and overall segmentation performance. However, U-Net and ON-DDU-Net show a performance advantage in the AJI, suggesting better instance segmentation quality. In the TNBC dataset, which exhibits greater heterogeneity within the same organ, NS-GUSL outperforms the other models in F1 and PQ metrics. As in the previous dataset, we observe that SwinU-Net and ON-DDU-Net perform better in the AJI and Dice metrics, pointing towards better instance separation. Across all external validation datasets, ON-DDU-Net achieves higher AJI scores, whereas NS-GUSL consistently obtains superior PQ, demonstrating its robust instance detection capability and overall panoptic segmentation quality.

### 5.2. Qualitative Results

**Figure 7:**
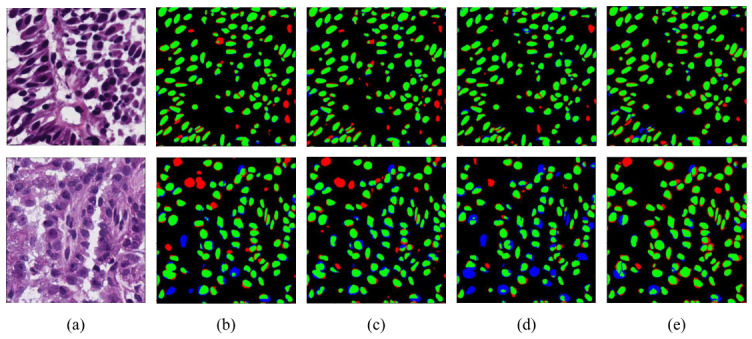
Qualitative comparison of results on the MoNuSeg dataset. Green—true positives, red—false positives, blue—false negatives. (**a**) Original image, (**b**) U-Net, (**c**) U-Net++, (**d**) SwinU-Net, and (**e**) NS-GUSL.

Figure 7 shows a qualitative comparison of the results of different methods on the MoNuSeg dataset. From the results on the MoNuSeg dataset, we note that NS-GUSL has relatively fewer false-positive and false-negative instances. This observation aligns with its high detection score, which indicates good recognition quality. On the other hand, a few methods perform slightly better on the segmentation end.

**Figure 8:**
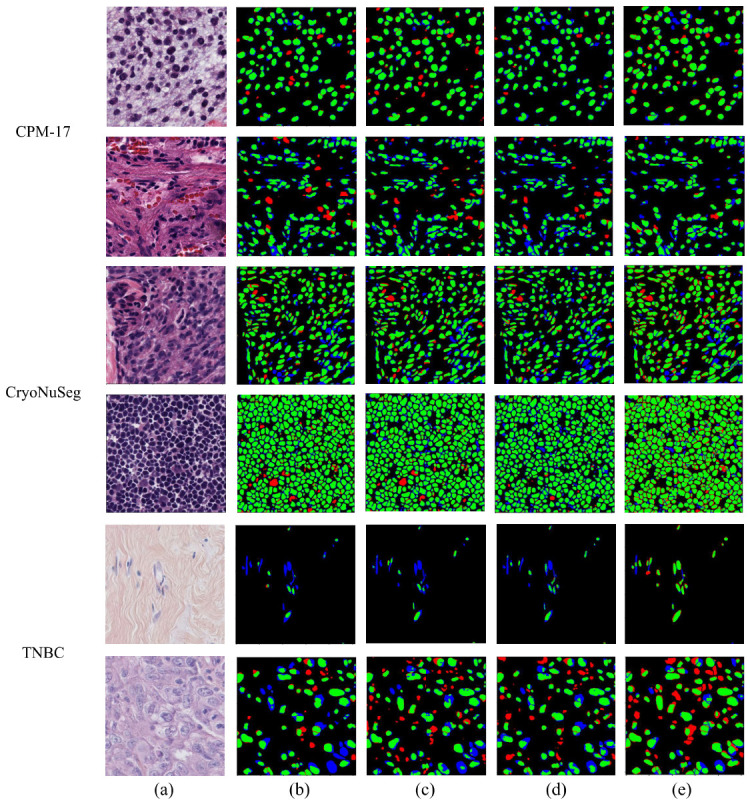
Qualitative comparison of results from external validation. Green—true positives, red—false positives, blue—false negatives. (**a**) Original image, (**b**) U-Net, (**c**) U-Net++, (**d**) SwinU-Net, and (**e**) NS-GUSL.

Figure 8 shows a qualitative comparison of the results of different models from the cross-dataset generalization study. From the first CPM-17 image, we can see that NS-GUSL has fewer false positives but misses some small, faintly stained nuclei instances. The second image shows regions falsely stained and predicted as nuclei by the other methods, but NS-GUSL correctly identifies them as background.

Most CryoNuSeg images have a dense distribution of nuclei of various shapes. Although most nuclei are detected in all four methods, they lack accurate boundary delineation and separation of overlapping nuclei. NS-GUSL uses the convex hull post-processing to split neighboring instances, and we observe cuts in some regions. The majority of these cuts are placed appropriately, but they sometimes split one instance into multiple smaller ones.

The overall appearance of TNBC images is considerably different from that of the other three datasets. These images have fewer nuclei, fainter stains, and lower contrast. In the first TNBC image, NS-GUSL captures the elongated instances that the other three methods fail to capture. In the second image, NS-GUSL captures more false positives, likely from regions with stains that pose as nuclei.

### 5.3. Ablation Study

To study the effectiveness of our proposed representation learning method, we compare it with deep neural network-based embedded learning methods. In order to provide a fair comparison, we only replace the representation learning module, and leave the remaining sections of the feature learning (i.e., feature selection and feature generation) and regression unchanged. We choose two popular networks, ResNet-50 and DenseNet-121, to learn embeddings. As NS-GUSL learns representations independently at each level, the DNN-based embeddings were also learned independently at each level. Further, we train these DNNs like an autoencoder with self-supervised learning, where the embeddings are learned by reconstructing the input. This is to ensure consistency with the PCA-based Saab transform that can reconstruct the input through an inverse transform. Only one layer of the DNNs is trained to get embeddings for the corresponding resolution, without the influence of other resolutions.

Table 7 shows the results of the comparison between the Saab transform features and the DNN features. We report the AJI, F1-score, Dice index, PQ, and the number of parameters required for representation learning at all levels. The Saab transform-based features outperform all DNN-based learned embeddings with just 319 parameters, while learning DNN embeddings requires millions of parameters. This study demonstrates the computational efficiency and superior performance of Saab-based features over the highly complex DNNs.

Table 8 presents the segmentation performance obtained using different low-confidence sample probability threshold ranges. As the interval widens, more samples are selected for LCSB. However, wider thresholds may also include samples with relatively high confidence predictions, thereby reducing the effectiveness of hard sample selection. The range (0.4, 0.6) achieves the best results across most performance metrics, providing a favorable trade-off between selecting uncertain samples and improving segmentation performance.

To evaluate the efficacy of binarization and post-processing techniques, we present the ablation study in Table 9. We compare simple global thresholding at 0.5 with the LCSB technique and analyze the impact of morphological post-processing in both cases.

The Dice index is stable in all variants, indicating a similar pixel-level overlap. With thresholding, our instance detection performance, measured by the F1 score, is the highest, due to fewer false negatives. Since PQ depends on both detection and segmentation, it is also high, as observed in the F1 and Dice scores. The LCSB module focuses on uncertain pixels, particularly in the boundary regions and some noisy or faintly stained instances. Due to this pixel-level operation, the improvement in AJI compared to thresholding is marginal, while we see a drop in the F1, Dice and PQ. The benefits of this module become more evident following the post-processing step that removes noisy predictions and fills holes. After these operations, all evaluation metrics improve, with particularly notable gains in AJI, F1, and PQ. The slightly lower detection score here comes from a lower recall due to missed predictions. The post-processing module considerably improves both detection and segmentation performance, particularly through better instance separation.

### 5.4. Model Size, Complexity, and Energy Consumption

In Table 10, we highlight the advantages of NS-GUSL in terms of model size, computational complexity, energy consumption, and carbon footprint for the prediction of one image patch. NS-GUSL has a very compact model, with fewer than a million parameters, while other DL models have more than 20 M parameters. For pixels with prediction probabilities outside the uncertain range, NS-GUSL requires only 17.26 K FLOPs per pixel, while NS-GUSL with the LCSB module requires 61.50 K FLOPs to label a pixel that has a prediction probability in the uncertain range. Despite the moderately higher complexity from LCSB, its advantage lies in its improved segmentation performance, as indicated by the AJI and PQ. U-Net and SwinU-Net require almost 7 times the FLOPs of NS-GUSL, while U-Net++ requires 16 times the FLOPs of the baseline NS-GUSL. The energy consumption can be computed from the FLOPs and model size, as suggested in [56], using the equation:

(8) $$\mathrm{Total}\;\mathrm{Energy}=E_{comp}\times \mathrm{FLOPs}+E_{mem}\times \mathrm{Model}\;\mathrm{Size}$$

where *E_comp_* is the cost of energy per floating-point operation, and *E_mem_* is the cost of energy to access one byte of memory. The hardware efficiency values are assumed to be constants and the same values are used to compute the energy for all models. In addition, we calculate the carbon footprint (in g CO_2_e) for all models with the following equation:

(9) CarbonFootprint=TotalEnergy×CarbonIntensity,

where carbon intensity is a location-based constant (e.g., 301 gCO_2_/kWh). Although U-Net++ and U-Net achieve good segmentation performance, they require 16 times and 7 times the energy of NS-GUSL, respectively, to achieve the same results. In particular, NS-GUSL uses only 14% of the energy of U-Net, making it a highly energy-efficient model for nuclei segmentation. In summary, NS-GUSL has a compact model, requires the fewest FLOPs/pixel, and yields the lowest energy consumption and carbon footprint as compared to the benchmarked DL models.

## 6. Discussion

From experimental analysis and benchmarking, NS-GUSL is shown to outperform DL models in multiple metrics on the MoNuSeg test set and the three external validation datasets. NS-GUSL also achieves superior detection scores in other datasets. It recognizes nuclei well despite their differences in size, shape, contrast, and stain intensity. In addition, NS-GUSL outperforms all methods in the panoptic quality metric. This shows its strong ability in panoptic segmentation, which jointly evaluates semantic and instance segmentation. NS-GUSL distinguishes between the nuclei and background classes and performs well in distinguishing between nuclei instances, resulting in a high PQ score.

Regarding the segmentation metric, the Dice index, NS-GUSL shows competitive performance on the MoNuSeg dataset and the external validation datasets compared to all DL methods. This suggests that, in addition to detecting nuclei, it segments them well and achieves a good pixel-level overlap between the ground truth and the prediction. However, the instance segmentation (AJI) results across external validation datasets show the limitations of the NS-GUSL model. This is mainly due to boundary errors and merged instances that penalize the AJI metric. At the pixel level, boundary regions are expected to have the highest degree of variability, stemming from staining variations, domain shifts, and subjective evaluation. Although the LCSB module aims to improve predictions in these low-confidence zones, highly confident, incorrect predictions continue to pose challenges. Future work could focus on better instance separation through a dedicated supervised boundary prediction path, or learned distance map-based representations that could be used to further refine nuclei contours.

Qualitative comparisons explain the behavior of the different models in images from different datasets. The NS-GUSL model effectively detects nuclei with irregular shapes and varying sizes when there is sufficient contrast between the foreground and background. In cases of low contrast and staining imperfections, NS-GUSL misses some instances entirely and falsely predicts others. Although the LCSB module identifies some of these challenging regions, they continue to cause difficulties. The preprocessing methods used in our work address these issues at the image level, whereas local variations are addressed through stain augmentations during the hard sample-aware training. Yet, the range of variations seen during training are limited, and can be improved with artifact- or noise-based augmentation, domain adaptation strategies, or test-time augmentation. In general, as shown in the quantitative and qualitative analyses of all datasets, NS-GUSL achieves a higher recall rate, indicating fewer missed detections than other methods. On the other hand, boundary predictions still require refinement to improve instance segmentation quality.

Although the performance of the models on the MoNuSeg test set is good, the decrease in the values of the performance metrics on the external validation datasets can be attributed to the domain shift arising from variations in staining procedures, acquisition protocols and patient populations. The models may be limited in their ability to capture the variability encountered during regular clinical practice. Our future work will focus on incorporating advanced stain normalization and domain adaptation techniques to further enhance the robustness and generalizability of our model.

In terms of model size and computational complexity, NS-GUSL has the smallest model size and requires less than 15% of the FLOPs needed by the baseline U-Net to compute the label for each pixel. Although the carbon footprint of SwinU-Net is comparable to that of NS-GUSL with LCSB, it requires a model that is 35 times larger. The minimal computational complexity and compact size of NS-GUSL enable seamless integration into the clinical workflow. In addition, it can be deployed on edge devices without requiring additional, expensive computational resources, such as GPUs, paving the way for sustainable AI solutions in healthcare.

## 7. Conclusions and Future Work

This work proposed an energy-efficient machine learning model, NS-GUSL, for nuclei segmentation. It features a U-shaped multi-scale architecture that successively refines the probability map from coarse to fine resolution through regression. The feed-forward, modular process enables a transparent, interpretable model for clinical applications. Evaluations on the MoNuSeg test set and generalization studies on the external validation datasets suggest its robust and competitive performance against DL methods. In addition, complexity analysis shows that NS-GUSL achieves competitive or superior performance to DL methods at lower cost and resource requirements, making it suitable for edge AI applications. In particular, it requires only 20% of the energy of the SwinU-Net to segment an image patch. In future research, we will include lightweight boundary-aware mechanisms to improve instance-level segmentation and study contrast enhancement strategies to enhance the detection of faintly stained instances, thus improving overall segmentation performance.

## Figures and Tables

**Table 1:** Neighborhood and Saab kernel sizes at different levels.

Level	Neighborhood Size	Saab Kernel Size
Level 1	9×9	3×3
Level 2	7×7	3×3
Level 3	5×5	3×3
Level 4	3×3	3×3

**Table 2:** Outline of feature number at each level.

Level/Module	Raw Features	RFT/DFT Selected Features	LNT Features	XGBoost Features
Level 4	375	375	137	512
Level 3	552	441	168	488
Level 2	648	518	247	612
Level 1	768	614	394	807
LCSB	535	155	205	238

**Table 3:** Results on the MoNuSeg Test Set. The best scores are shown in **bold**, and the second-best scores are underlined.

Method	AJI	F1	Dice	PQ
U-Net [12]	0.5668	0.8463	0.7838	0.7364
SwinU-Net [33]	0.5812	0.8162	0.7862	0.6937
U-Net++ [32]	0.5877	0.8339	0.7947	0.7419
CMF-UNet [54]	0.6153	0.8226	-	-
UCTransNet [55]	0.4652	0.8709	0.7986	0.6470
ON-DDU-Net [39]	**0.6620**	-	**0.8320**	0.6357
NS-GUSL (Ours)	0.6060	**0.8849**	0.7948	**0.7727**

**Table 4:** External Validation Results: MoNuSeg (Train) → CryoNuSeg (Validation). The best scores are shown in **bold**, and the second-best scores are underlined.

Method	AJI	F1	Dice	PQ
U-Net [12]	0.4778	**0.8221**	0.7779	0.5876
SwinU-Net [33]	0.4825	0.7840	0.7634	0.5510
U-Net++ [32]	0.4676	0.8061	0.7607	0.5931
ON-DDU-Net [39]	**0.5040**	-	**0.7890**	0.4710
NS-GUSL (Ours)	0.4500	0.8129	0.7538	**0.6177**

**Table 5:** External Validation Results: MoNuSeg (Train) → CPM-17 (Validation). The best scores are shown in **bold**, and the second-best scores are underlined.

Method	AJI	F1	Dice	PQ
U-Net [12]	0.5662	0.8415	0.7883	0.5659
SwinU-Net [33]	0.5419	0.8223	0.7697	0.5009
U-Net++ [32]	0.5661	0.8390	0.7949	0.5758
ON-DDU-Net [39]	**0.6380**	-	**0.8200**	0.5890
NS-GUSL (Ours)	0.5479	**0.8723**	0.8016	**0.6106**

**Table 6:** External Validation Results: MoNuSeg (Train) → TNBC (Validation). The best scores are shown in **bold**, and the second-best scores are underlined.

Method	AJI	F1	Dice	PQ
U-Net [12]	0.4561	0.7180	0.6734	0.4713
SwinU-Net [33]	0.5052	0.7569	0.7107	0.4969
U-Net++ [32]	0.4973	0.7409	0.6975	0.5130
ON-DDU-Net [39]	**0.6080**	-	**0.7840**	0.5810
NS-GUSL (Ours)	0.4602	**0.7806**	0.6965	**0.7383**

**Table 7:** Ablation study of representation learning on the MoNuSeg test set. The best scores and fewest parameters are shown in **bold**.

Representation Learning Method	AJI	F1	Dice	PQ	Parameters
ResNet-50	0.5265	0.7164	0.7857	0.6719	1.75M
DenseNet-121	0.5617	0.6028	0.7871	0.6040	2.71M
Saab Transform	**0.5840**	**0.8587**	**0.7942**	**0.6841**	**319**

**Table 8:** Comparison of segmentation results for different low-confidence sample (LCS) probability thresholds. The best scores are shown in **bold**.

LCS Probability Thresholds	AJI	F1	Dice	PQ
(0.1, 0.9)	0.4950	0.6996	0.7760	0.6200
(0.2, 0.8)	0.5320	0.7602	0.7885	**0.6852**
(0.3, 0.7)	0.5550	0.7319	0.7932	0.6541
(0.4, 0.6)	**0.5840**	**0.8587**	**0.7942**	0.6841

**Table 9:** Ablation study of binarization and post-processing techniques on the MoNuSeg test set. The checkmark (✓) indicates the applied modules. The best scores are shown in **bold**.

Thresholding (0.5)	LCSB	Post-Processing	AJI	F1	Dice	PQ
✓			0.5818	0.8852	**0.8010**	0.7227
✓		✓	0.5994	**0.8911**	0.7929	**0.7978**
	✓		0.5840	0.8587	0.7942	0.6841
	✓	✓	**0.6060**	0.8849	0.7948	0.7727

**Table 10:** Comparison of model size, FLOPs/pixel, energy and carbon footprint. The best values are shown in **bold**.

Method	Model Size	FLOPs/Pixel	Energy ($10^{-7}$ kWh)	Carbon Footprint ($10^{-5}$ g CO_2_e)
SwinU-Net [33]	27 M (×35)	118 K (×7)	8.29 (×5)	24.9 (×5)
U-Net++ [32]	26 M (×34)	281 K (×16)	25.7 (×16)	77.3 (×16)
U-Net [12]	24 M (×32)	120 K (×7)	10.9 (×7)	33 (×7)
NS-GUSL (with LCSB)	0.8 M (×1)	61.50 K (×4)	5.60 (×4)	16.8 (×4)
NS-GUSL	**0.76 M (×1)**	**17.26 K (×1)**	**1.57 (×1)**	**4.73 (×1)**

## Data Availability

Publicly available datasets were analyzed in this study. The MoNuSeg dataset is available at https://monuseg.grand-challenge.org/Data/ (accessed on 2 May 2026). The CryoNuSeg dataset is available at https://www.kaggle.com/datasets/ipateam/segmentation-of-nuclei-in-cryosectioned-he-images (accessed on 5 May 2026). The CPM-17 and TNBC datasets are available at https://drive.google.com/drive/folders/1l55cv3DuY-f7-JotDN7N5nbNnjbLWchK (accessed on 5 May 2026).
